# Biomonitoring and Elimination of Perfluorinated Compounds and Polychlorinated Biphenyls through Perspiration: Blood, Urine, and Sweat Study

**DOI:** 10.1155/2013/483832

**Published:** 2013-09-03

**Authors:** Stephen J. Genuis, Sanjay Beesoon, Detlef Birkholz

**Affiliations:** ^1^Environmental Health Sciences, University of Alberta, 2935-66 Street, Edmonton, AB, Canada T6K 4C1; ^2^Department of Laboratory Medicine, University of Alberta, Edmonton, AB, Canada T6G 2B7; ^3^Environmental Division, A.L.S. Laboratory Group, Edmonton, AB, Canada T6E 5C1

## Abstract

Perfluorinated compounds (PFCs) are man-made organofluorine chemicals manufactured and marketed for their stain-resistant properties. Polychlorinated biphenyls (PCBs) are anthropogenic organochlorine compounds previously used in various industrial and chemical applications prior to being banned in the Western world in the 1970s. Both PFCs and PCBs are persistent contaminants within the human organism and both have been linked to adverse health sequelae. Data is lacking on effective means to facilitate clearance of PFCs and PCBs from the body. *Methods*. Blood, urine, and sweat were collected from 20 individuals (10 healthy participants and 10 participants with assorted health problems) and analyzed for PFCs and PCBs using high performance liquid chromatography tandem mass spectrometry. *Results*. Some individual PCB congeners, but not all, were released into sweat at varying concentrations. None of the PFCs found in serum testing appeared to be excreted efficiently into perspiration. *Conclusions*. Induced perspiration may have some role in facilitating elimination of selected PCBs. Sweat analysis may be helpful in establishing the existence of some accrued PCBs in the human body. Sweating does not appear to facilitate clearance of accrued PFHxS (perfluorohexane sulfonate), PFOS (perfluorooctane sulfonate), or PFOA (perfluorooctanoic acid), the most common PFCs found in the human body.

## 1. Background

Following the advent of sophisticated tools of production and analytical technologies to characterize individual chemicals after the Second World War, an unprecedented upsurge in the diversity and production volume of chemical agents occurred throughout the second half of the 20th and the early part of the 21st century. Applications for assorted chemicals eventually came to include a range of goods and services from use in industrial processes to inclusion in regular consumer products such as food, clothing, electronics, cleaning products, and cosmetics. As a result, synthetic chemicals and various by-products are now integrated into most aspects of our daily lives. Adverse sequelae of the chemical revolution have recently been recognized, with several studies confirming that many chemical compounds have accumulated and persist within the environment and within people [1, 2], with attendant health sequelae in many cases [3, 4]. In response, research is currently underway to determine methods to eliminate persistent compounds from the body in order to preclude and overcome health problems [5, 6]. 

Although there are millions of chemical compounds available around the globe [7], only a fraction of these are regulated under specific legislation in key markets [8]. Examples of regulatory codes include the Toxic Substances Control Act (TSCA) in the United States, the Domestic Substances List (DSL) in Canada, and the Registration, Evaluation, Authorization, and Restriction of Chemical substances Act (REACH) in Europe. Despite a battery of policies, regulations, and laws around the world, most human populations are chronically exposed to significant levels of diverse environmental toxicants [1, 6]. This is likely due to (i) lack of regulation in some jurisdictions, (ii) noncompliance with existing regulations, (iii) insufficient data on many chemicals to inform regulatory policy, and (iv) lack of political will to enforce standards in some locales. Following the creation of the United States Environmental Protection Agency (USEPA) in 1970 as a Federal regulatory agency, many other countries in the Western hemisphere have followed suit and created regulatory bodies to monitor the environment and protect human health.

Considerable effort and resources have recently been dedicated to research on toxicity of environmental chemicals, exposure science, and human epidemiology studies looking at exposure-disease associations between chemicals and health outcomes. Nevertheless, there has been little recognition that regulations and public education, aimed at minimizing human exposure to and uptake of some harmful chemical toxicants, are not enough to address the already present “internal dose” of toxicants harbored within many in the general population [9], including neonates [10]. Based on the basic epidemiologic model of causation involving host, agent, and environment, there is a need for the medical research community to focus on efficient ways of clearing the host of the agent, in this case the environmental toxicants [6]. The same principle is used in infectious disease epidemiology, whereby in addition to limiting the human exposure to pathogens, research has led to the discovery of antibiotics, antivirals, and antiparasitic medications to eradicate the source pathogen.

In a recent review, Sears and Genuis [11] proposed a three-pronged approach to decrease the body burden of environmental chemical toxicants in humans, namely, by avoiding exposure, by maintaining the integrity of organs involved in detoxification (mainly the liver, lungs, and kidneys), and finally by intervening directly on the body to catalyse the excretion of these compounds. Interventions to enhance the rapid excretion of xenobiotics can occur by varying means including administering selected pharmaceuticals known to bind toxicants and facilitate their elimination through urine or feces, by pulmonary excretion evident with intense exercise, by blood purification techniques including dialysis and plasmapheresis, and by thermal depuration—or induced sweating of toxicants out through the skin [5, 12].

An easily accessible method to facilitate removal of a broad range of environmental chemicals is through induced perspiration [11, 13–17]. Efficacy of this mode of xenobiotic excretion is not surprising given the fact that multiple drugs can be reliably detected in human sweat samples [18–21]. The fluid evident in perspiration appears to contain both hydrophilic compounds as a result of the watery nature of the exudate from sweat glands and lipophilic compounds as a result of the oily nature of the exudate from sebaceous glands. Accordingly, it is postulated that perhaps detoxification through induced perspiration may be effective for many compounds as persistent hydrophilic compounds may be released into sweat while persistent lipophilic compounds may be emitted into sebum.

This paper is part of a series, wherein we report the outcomes of research comparing the elimination of a spectrum of chemical toxicants in urine versus sweat in relation to their concentration in serum. We reported previously on the elimination of many metals [22], BPA [23], and phthalates [24] through induced perspiration. In this paper we compare the elimination kinetics of perfluorinated compounds (PFCs) and polychlorinated biphenyls (PCBs) into urine and sweat.

### 1.1. Perfluorinated Compounds (PFCs)

PFCs are a family of man-made chemicals with many applications including repelling stains on furniture, carpets, and clothing as well as providing a barrier to prevent adherence in food packaging and nonstick cooking surfaces. These compounds structurally consist of a linear or branched carbon backbone that is entirely substituted by strong bonds to fluorine atoms. The fluorine component of PFCs provides extremely low surface tension and accounts for their unique hydrophobic (water repelling) and lipophobic (lipid repelling) nature [25]. As many PFCs are very stable, nonreactive, and effective at low concentrations, they have been used to make commercial products that are resistant to both water and oil, that are stain resistant and that can also withstand the extremes of temperature, pH, and oxidizing conditions.

In recent years, studies have demonstrated that PFC bioaccumulation is a common event [26], and research has linked assorted health concerns to exposure and accrual of PFCs, including the sequelae of gestational PFC contamination. In addition, animal and human studies have now linked PFC exposure with developmental toxicity [27], neurotoxicity [28], reproductive toxicity [29], cardiovascular toxicity [26], metabolic dysregulation [30], development of arthritis [31], carcinogenesis [32], and immunotoxicity [33]. There has been minimal research exploring strategies to eliminate PFCs [34]. 

### 1.2. Polychlorinated Biphenyls (PCBs)

PCBs are anthropogenic organic chlorine chemicals composed of 2 benzene rings (thus the name biphenyl) with up to 10 chlorine atoms attached to the rings. The general formula can be written as C_12_H_10−*X*_Cl_*X*_. Depending on the number, position, and attachment of the chlorine atoms on the biphenyl rings, 209 different configurations (PCB congeners) are possible. Given their unique chemical and physical properties such as excellent chemical stability, high dielectric constants, and thermal conductivity, they have been extensively used in hundreds of industrial and chemical applications such as coolants and insulators in transformers, capacitors, and motors. Furthermore they are used as plasticizers in paints and coatings of electrical wires and other electronic parts. However, based on data showing environmental persistence and probable adverse effects on wildlife and humans, PCBs were banned in the western world in the late 1970s and were later classified in 2001 as persistent organic pollutants by the “Stockholm Convention on Persistent Organic Pollutants.”

Among the most notable adverse human health effects of PCBs are endocrine disruption [35, 36], hypertension [37], cutaneous malignant melanoma [38], and non-Hodgkin's Lymphoma [39]. Given that PCBs are lipophilic compounds, they tend to accumulate in human adipose tissue and unsurprisingly have long elimination half-lives, ranging from 4 to 9 years [40, 41] depending on the congener and exposure level. Data is lacking in the medical literature, however, on effective means to facilitate PCB elimination.

## 2. Methods

### 2.1. Participant Recruitment

9 males and 11 females with mean ages 44.5 ± 14.4 years and 45.6 ± 10.3 years, respectively, were recruited to participate in this study after appropriate ethical approval was received from the Health Research Ethics Board of the University of Alberta. 10 participants were patients with various clinical conditions, and 10 were otherwise healthy adults. Participants with health issues were recruited from the first author's clinical practice by invitation, and both healthy and sick individuals were selected as samples of convenience by availability, wish to participate, and ease of contact. Each participant in the study provided informed consent and volunteered to give one 200 mL random sample of blood, one sample of first morning urine and one 100 mL sample of sweat. Demographic and clinical characteristics of all research participants are provided in Table 1.

### 2.2. Samples Collection

All blood samples were collected at one DynaLIFE laboratory site in Edmonton, Alberta, Canada, with vacutainer blood collection equipment (BD Vacutainer, Franklin Lakes, NJ, USA) using 21-gauge stainless steel needles which were screwed into the “BD Vacutainer One-Use Holder” (REF 364815). The 10 mL glass vacutainer was directly inserted into the holder and into the back end of the needle. This process and the use of glass blood collection tubes were used to prevent contamination. Blood was collected directly into plain 10 mL glass vacutainer tubes, allowed to clot, and after 30 minutes were centrifuged for 10 minutes at 2,000 revolutions per minute (RPM). After serum was separated off, samples were picked up by ALS Laboratories (about 3 kilometres from the blood collection site) for storage pending analysis. When received at ALS, serum samples were transferred to 4 mL glass vials and stored in a freezer at −20°C, pending transfer to the analytical laboratory.

For urine collection, participants were instructed to collect a first morning midstream urine sample directly into a provided 500 mL glass jar container on the same day blood samples were collected. Urine samples were delivered by the participants directly to ALS Laboratories, Edmonton. Samples were transferred to 4 mL glass vials and stored in a freezer at −20°C, pending transfer. 

For sweat collection, participants were instructed to collect perspiration from any site on their body directly into the provided 500 mL glass jar container—by placing the jar against their prewashed skin (with toxicant-free soap, water, and nonplastic brush) when actively sweating or by using a stainless steel spatula against their skin to transfer perspiration directly into the glass jar. (Stainless steel—made up primarily of iron, chromium, and nickel—was chosen as it is the same material as the needles used in standard blood collections and is reported not to off-gas or leach at room or body temperature.) In excess of 100 mL of sweat was provided in all but one case.

Each of the glass bottles used for sampling in this study was provided by ALS Laboratories and had undergone extensive cleaning and rinsing. The containers were deemed appropriate for sweat collection with negligible risk of contamination: laboratory-grade phosphate-free detergent wash; acid rinse; multiple hot and cold deionized water rinses; oven dried; and capped and packed in quality-controlled conditions. Sweat was collected within 1 week before or after collecting the blood and urine samples. No specifications were given as to how long sweating had commenced before collection. 10 participants collected sweat inside a dry infrared sauna, 7 collected inside a steam sauna, and 3 collected during and immediately after exercise—no specific instruction was given regarding the type or location of exercise. Participants were educated about the research and were asked to meticulously avoid exposure to any potential sources of toxicants around the time of collection.

Sweat was delivered by the participants directly to ALS Laboratories. Samples were transferred to 4 mL glass vials and stored in a freezer at −20°C, pending analysis. No preservatives were used in neither the jars provided for sweat and urine collection nor in the serum storage vials. PFC and PCB analysis for each sample was performed by liquid chromatograph tandem mass spectrometry using multiple reaction monitoring. Testing was undertaken for levels of 7 PFCs: (i) PFHxS (perfluorohexane sulfonate), (ii) PFOS (perfluorooctane sulfonate), (iii) PFOA (perfluorooctanoic acid), (iv) PFNA (perfluorononanoic acid), (v) PFDA (perfluorodecanoic acid), (vi) PFUA perfluoroundecanoic acid, and (vii) PFTA perfluorotetradecanoic acid. Testing was done for 35 congeners of PCBs.

## 3. Results and Discussion

**Figure 1:**
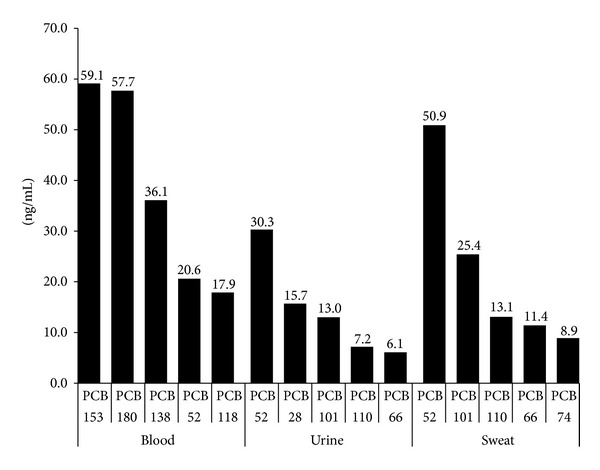
Mean blood, urine, and sweat concentrations of specific PCB congeners.

**Figure 2:**
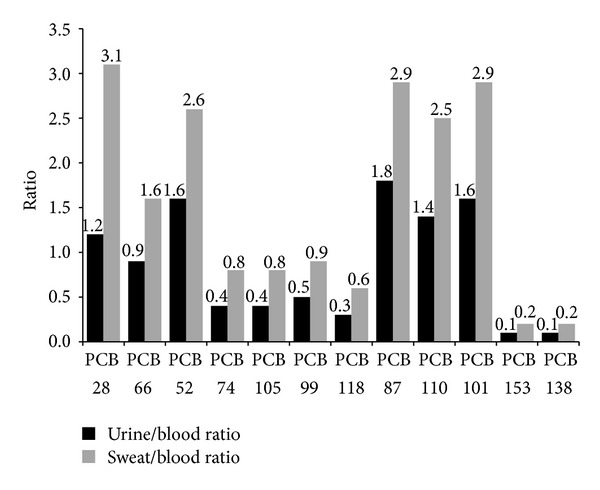
Mean urine/blood ratio and sweat/blood ratio for specific PCB congeners.

Participant demographics and general clinical characteristics are provided in Table 1. Mean blood, urine, and sweat concentrations of specific PCB congeners are provided in Figure 1 while Figure 2 displays the mean urine/blood ratio and sweat/blood ratio for specific PCB congeners.

### 3.1. Results of Elimination of Perfluorinated Compounds (PFCs)

4 of the 20 study subjects had PFHxS serum levels above the 95th percentile reported in the NHANES Study [9]. The same 4 of the 20 study participants had serum levels of PFOS that were above the 90th percentile in the NHANES Study and PFOA levels above the 50th percentile of the NHANES group. PFNA levels for the same 4 of 20 subjects were present but well below the mean NHANES PFNA values. In all 20 participants, the serum levels of all remaining PFCs were very low to none detected.

Despite finding considerable amounts of PFHxS, PFOS, and PFOA in the serum of 20 percent of the participants, minimal to none of any PFC tested was detected in either the sweat or the urine of any of the participants. It is clearly evident that induced perspiration through sauna or exercise does not seem to hasten the clearance of these three common PFCs from the human body via perspiration.

### 3.2. Results of Elimination of Polychlorinated Biphenyls (PCBs)

Of the 35 PCB congeners tested for, three main PCBs were detected in our cohort of 20 adults: congeners 153, 180, and 138. This finding appears to be in line with what is previously reported in the literature [42, 43]. PCBs 52, 101, 110, and 66 were detected at fairly high levels in both urine and sweat. The relatively higher levels in sweat suggest that perspiration may be a more efficient method of excreting these congeners, compared to urinary elimination.

In an effort to compare the relative elimination efficiencies of the different PCBs, we calculated the urine/blood (U/B) and sweat/blood (S/B) ratios for those congeners that are detectable in both body fluids. A ratio of 1 indicates a similar concentration in blood and the body fluid investigated (urine or sweat), a ratio below one indicates a higher concentration in blood, and a ratio above 1 indicates a higher concentration in the body fluid than in blood. Data presented in Figure 2 show that the mean S/B ratios are consistently higher than the U/B ratios for all the congeners presented, which is suggestive that sweat may be a potentially significant elimination pathway for some PCBs. 

Interestingly, for two of the three major PCBs detected in the blood of the 20 participants, namely, PCBs 153 and 138, both the U/B and the S/B ratios are low, suggesting that these compounds are poorly excreted, thus explaining their higher bioaccumulative potential and why these congeners may be found more readily in population blood testing. PCB 180 was detected in 9 urine samples, with the U/B ratios ranging from 0.01 to 0.17, and in 14 sweat samples, with the S/B ratios ranging from 0.02 to 0.15. It is unclear why there is such a marked difference in excretion rates in perspiration between different PCB congeners. In review, recognizing the persistent nature of many PCBs in the human body, induced perspiration through means such as regular sauna therapy may have some role as a clinical modality to facilitate the clearance of some but not all PCB compounds. 

## 4. Conclusion

Emerging research continues to confirm that, after environmental exposure, many chemical toxicants persist as contaminants within the human body and many persistent compounds have recently been correlated with adverse health outcomes. With the current recognition that up to 90% of ongoing illness may be related to environmental determinants [3], focused attention is being applied to the challenge of ubiquitous chemical exposures. Emerging evidence continues to demonstrate that, in the early 21st century, most people already carry an existing internal dose or body burden of persistent chemical pollution [9]. This realization has prompted some scientists to explore interventions to detoxify or to facilitate bioelimination of persistent contaminants in order to preclude and overcome illness [6].

Various studies now confirm that depuration through induced perspiration may be an effective way to facilitate human clearance of many persistent pollutants from within the body [15, 17, 22]. This study demonstrates, however, that the perfluorinated compounds PFHxS, PFOS, and PFOA are not effectively eliminated in perspiration. On the other hand, induced perspiration appears to be successful at increasing the clearance of some but not all PCB congeners.

As many persistent pollutants are stored primarily within tissues, however, serum levels of PCBs may not adequately reflect total bioaccumulated PCB levels within the body. Although favorable, even a 2 : 1 or 3 : 1 Sweat/Blood elimination ratio with induced perspiration for some PCBs, as was found in this study, may represent a modest removal rate when exploring effective clinical interventions to rapidly eliminate the total body burden of PCBs from individuals poisoned with these toxicants. Accordingly, additional clinical modalities to facilitate elimination of PFC and PCB compounds will likely be required in therapeutic settings [5, 12, 34]. Further study is needed to explore and quantify the scale of removal of various persistent toxic substances via induced perspiration and other detoxification interventions in relation to total body burden.

*Key Points*

- Induced perspiration does not seem to hasten the clearance of any of the common PFCs (perfluorinated compounds)—PFHxS (perfluorohexane sulfonate), PFOS (perfluorooctane sulfonate), or PFOA (perfluorooctanoic acid)—from the human body.
- Induced perspiration does appear to hasten the elimination of some, but not all, PCB (polychlorinated biphenyls) congeners from the human body.

## Figures and Tables

**Table 1:** Participant demographics and general clinical characteristics.

Participant	Gender	Age	Clinical diagnosis	Technique used for sweat collection
1	M	61	Diabetes, obesity, and hypertension	Exercise
2	F	40	Rheumatoid arthritis	Steam Sauna
3	M	38	Addiction disorder	Steam Sauna
4	F	25	Bipolar disorder	Steam Sauna
5	F	47	Lymphoma	Steam Sauna
6	F	43	Fibromyalgia	Steam Sauna
7	F	48	Depression	Steam Sauna
8	F	40	Chronic fatigue	Infrared Sauna
9	F	68	Diabetes, fatigue, and obesity	Steam Sauna
10	M	49	Chronic pain, cognitive decline	Exercise
11	M	53	Healthy	Exercise
12	M	23	Healthy	Infrared Sauna
13	M	21	Healthy	Infrared Sauna
14	F	47	Healthy	Infrared Sauna
15	M	53	Healthy	Infrared Sauna
16	F	43	Healthy	Infrared Sauna
17	F	51	Healthy	Infrared Sauna
18	M	46	Healthy	Infrared Sauna
19	M	57	Healthy	Infrared Sauna
20	F	50	Healthy	Infrared Sauna
